# Effective photochemical treatment of a municipal solid waste landfill leachate

**DOI:** 10.1371/journal.pone.0239433

**Published:** 2020-09-22

**Authors:** Ardak Makhatova, Birzhan Mazhit, Yerbol Sarbassov, Kulyash Meiramkulova, Vassilis J. Inglezakis, Stavros G. Poulopoulos

**Affiliations:** 1 Department of Chemical and Materials Engineering, School of Engineering and Digital Sciences, Environmental Science & Technology Group (ESTg), The Environment & Resource Efficiency Cluster (EREC), Nazarbayev University, Nur-Sultan, Kazakhstan; 2 Department of Environmental Engineering and Management, Faculty of Natural Sciences, L.N.Gumilyov Eurasian National University, Nur-Sultan, Kazakhstan; 3 Department of Chemical and Process Engineering, University of Strathclyde, Glasgow, United Kingdom; Universiti Malaysia Pahang, MALAYSIA

## Abstract

This work aimed at studying the photochemical treatment of a landfill leachate using ultraviolet light, hydrogen peroxide, and ferrous or ferric ions, in a batch recycle photoreactor. The effect of inorganic carbon presence, pH, initial H_2_O_2_ amount (0–9990 mg L^-1^) as well as Fe(II) (200–600 ppm) and Fe(III) (300–700 ppm) concentrations on the total carbon removal and color change was studied. Prior to the photochemical treatment, a pretreatment process was applied; inorganic nitrogen and inorganic carbon were removed by means of air stripping and initial pH regulation, respectively. The leachate sent subsequently for photochemical treatment was free of inorganic carbon and contained only organic carbon with concentration 1200±100 mg L^-1^ at pH 5.1–5.3. The most favorable concentrations of H_2_O_2_ and ferric ions for carbon removal were 6660 mg L^-1^ and 400 ppm, respectively. Adjusting the initial pH value in the range of 2.2–5.3 had a significant effect on the organic carbon removal. The photo-Fenton-like process was more advantageous than the photo-Fenton one for leachate treatment. By applying the most favorable operating conditions, 88.7% removal of total organic carbon, 100% removal of total inorganic carbon, 96.5% removal of total nitrogen, and 98.2% color removal were achieved.

## Introduction

Due to its economic advantage, sanitary landfilling remains the most widely used method for the disposal of municipal solid waste (MSW) in most countries [1]. Approximately 125 m^3^ of greenhouse emissions composed of 65% of methane and 34% of carbon dioxide are released per ton of landfilled MSW [2]. Moreover, a dark, highly toxic with unpleasant smell liquid results from the disintegration of the organic matter of the waste and the infiltration of rainwater through landfills, which is called landfill leachate. It contains high amounts of organic recalcitrant compounds [3] as well as chlorinated organic and inorganic salts, heavy metals, and elevated levels of ammonia-nitrogen [1].

Untreated leachates can cause soil, groundwater and surface water pollution [4]. Refractory organic components, ammonia and heavy metals are the major concerns associated with the landfill leachate [4]. Specifically, ammonia in excess concentration is toxic to aquatic organisms [5], while heavy metals can be harmful even at low concentrations [6]. Refractory organic compounds include pesticides and herbicides, substituted phenols, surfactants, and non-biodegradable chlorinated solvents [7]. The environmental hazard of landfill leachate in water has been confirmed by toxicity analyses conducted using various test organisms (Daphnia similes, Brachydanio rerio, etc.) [1].

The leachate composition depends largely on the landfill age [8]. When the landfill age exceeds the 10 years, the leachate is considered mature [2, 4]. In mature (stabilized) leachates, there is a methanogenic phase, in which growth of methanogenic microorganisms in the waste and transformation of free volatile fatty acids (VFAs) to biogas (CH_4_, CO_2_) take place [9]. An acidogenic phase takes place in young leachates, which leads to the formation of anaerobic fermentation products including large amounts of VFAs, such as acetic acid, propionic acid and butyric acid [10]. As a result, young leachates contain high quantities of biodegradable organic compounds [11], while as leachates mature refractory (non-biodegradable) substances like humic and fulvic acids dominate the organic fraction [9]. Biological processes are effective in the treatment of young landfill leachates due to the high values of BOD_5_/COD (Biochemical Oxygen Demand/Chemical Oxygen Demand) ratio involved [12]. In contrast, mature leachates exhibit low BOD_5_/COD ratio and relatively high NH_3_-N concentration [1], which dictates the necessity of the use of physicochemical treatment to remove the less biodegradable compounds [4]. Collecting and treating the generated leachate can prevent the pollution of ground and surface water, protecting thus public health and ecosystems [2].

Various advanced oxidation processes (AOPs) have been applied for the treatment of landfill leachates [13]. AOPs is a term used to describe a variety of chemical methods that have been proved efficient for the elimination of hazardous organic molecules in water [14]. The efficiency of AOPs depends on reactive oxidative agents [15], such as hydroxyl radicals (•OH), which are the most common choice as they are highly active [16]. Hydroxyl radicals attack almost all organic compounds indiscriminately and react 10^6^−10^9^ times faster than other oxidizing agents like ozone [17]. Eq (1) represents the general oxidation process of organic matter (R-H) by •OH radical via removing protons. The resulting organic radicals (•R) can be easily further oxidized [18]:

$$R-H+{HO}^{•}\to H_{2}O+R^{•}\to further\;oxidation.$$ (1)

The hydroxyl radicals can be obtained by photochemical methods, such as UV/H_2_O_2_, UV/O_3_, UV/O_3_/H_2_O_2_, photo-Fenton/Fenton-like systems, and photocatalytic oxidation [19]. In cases of highly concentrated wastewaters like leachates, the classical oxidation by O_3_ or H_2_O_2_ does not decompose easily organic compounds to CO_2_ and H_2_O [20, 21], although it is theoretically possible given time [22]. Moreover, the oxidation intermediates remaining in the reaction mixture can be even more harmful than the parent compounds [19].

The Fenton method is among the most widely applied AOPs, in which the Fenton’s reagent, a mixture of a ferrous salt (catalyst) and hydrogen peroxide (oxidant), is used to destroy refractory organic compounds [23]. The Fenton reaction depends on the production of hydroxyl radicals and it is considered as a convenient and economical process [24]. The Fenton mechanism is described by the following reactions when no organic compounds are present in the solution [4]:

$${Fe}^{2+}+H_{2}O_{2}\to {Fe}^{3+}+{OH}^{•}+{OH}^{-}$$ (2)

$${Fe}^{3+}+H_{2}O_{2}\to {Fe}^{2+}+{HO}_{2}^{•}+H^{+}$$ (3)

$${OH}^{•}+H_{2}O_{2}\to {HO}_{2}^{•}+H_{2}O$$ (4)

$${OH}^{•}+{Fe}^{2+}\to {Fe}^{3+}+{OH}^{-}$$ (5)

$${Fe}^{3+}+{HO}_{2}^{•}\to {Fe}^{2+}+O_{2}H^{+}$$ (6)

$${Fe}^{2+}+{HO}_{2}^{•}+H^{+}\to {Fe}^{3+}+H_{2}O_{2}$$ (7)

$$2{HO}_{2}^{•}\to H_{2}O_{2}+O_{2}.$$ (8)

The Eq (2) occurs in acidic conditions. The chemical Eq (3) between ferric ions (Fe^3+^) and H_2_O_2_ is known as Fenton-like reaction [25].

The classical Fenton reaction (Eq 2) under UV irradiation is called photo-Fenton (Eq 9). The light source enhances the catalytic reduction of Fe^3+^ into Fe^2+^ in H_2_O_2_ aqueous solutions, thereby increasing the generation of •OH radicals:

$${Fe}^{3+}+H_{2}O+h\nu \to {Fe}^{2+}+H^{+}+{OH}^{•}.$$ (9)

A large formation of Fe^3+^ species in the classical Fenton reaction (Eq 2) slows down the treatment efficiency. This disadvantage is eliminated in the photo-Fenton process, as the reductive photolysis of [Fe(OH)]^2+^ (Eq 10) regenerates the Fe^2+^ ions that catalyze the Fenton reaction and provide additional •OH radicals [26]:

$${Fe(OH)}^{2+}+h\nu \to {Fe}^{2+}+{OH}^{•}.$$ (10)

Ultraviolet irradiation in the photo-Fenton process can also directly generate •OH through the decomposition of hydrogen peroxide [27]. Several UV regions can be used by the photo-Fenton process as a source of light energy, specifically UVA (*λ* = 315–400 nm), UVB (*λ* = 285–315 nm), and UVC (*λ <* 285 nm). The intensity and wavelength of UV radiation has a significant influence on the decomposition rate of organic pollutants [27]. A more detailed reaction mechanism of the Fenton process under acidic pH has been provided by De Laat and Gallard [28].

The photo-Fenton process can significantly improve the leachate characteristics in terms of organic carbon contained, color and odor. Moreover, toxic organic matter can be removed, and the biodegradability of organic substances can be increased [4]. The application of the photo-Fenton process to treat landfill leachates has been reported in several cases [29–32]. Primo et al. [29] studied several oxidation processes and their efficiency in removing organic substances from landfill leachates, and they stated that the photo-Fenton process was the most effective among the ones studied. The removal efficiency obeyed the following sequence: photo-Fenton > Fenton-like > Fenton > UV/H_2_O_2_ > UV alone. Hermosilla et al. [30] compared Fenton and photo-Fenton processes in the treatment of a landfill leachate (Madrid, Spain). By the photo-Fenton treatment, 32 times less iron was used to achieve the same COD removals as with the conventional Fenton treatment, which resulted in 25 times less iron sludge volume produced. Kim and Huh [32] observed additional 35–50% total organic carbon removals using photo-Fenton instead of the conventional Fenton process during the treatment of a leachate.

Many attempts have been made in the direction of treating landfill leachates, and the favorable operating conditions for photo-Fenton processes are known, however no absolute recommendations that can be directly applied universally exist [29]. More efforts are still required in the study of the photochemical treatment of real highly concentrated wastewaters like leachates, the management of which pose many challenges. Specifically, the variation in leachate composition from one landfill to another and with time makes each study a different case. Currently, the leachate generated at the municipal solid waste landfill of the city of Nur-Sultan is left untreated.

The aim of this study was to employ photo-Fenton and photo-Fenton-like methods for the effective treatment in terms of carbon and color removal of a mature leachate from the municipal solid waste landfill of Nur-Sultan (Kazakhstan). The efficiency of photochemical processes is strongly connected to the initial amounts of reactants used. Therefore, the effect of inorganic carbon presence, initial concentrations of ferrous ions (200–600 ppm), ferric ions (300–700 ppm), hydrogen peroxide amount (0–9990 mg L^-1^), and of the initial pH value on the total organic carbon (TOC) removal and color change of the landfill leachate were investigated to identify the most favorable operating conditions. Air stripping and pH adjustment were conducted prior to the photochemical treatment to remove ammonia and inorganic carbon from the leachate.

## Materials and methods

### Chemicals

Hydrochloric acid (37% w/w, Sigma-Aldrich) or sodium hydroxide solution (10 M) prepared from sodium hydroxide (pellets) purchased from Fisher Chemical was used for pH adjustment. Specifically, 0.046 mL of HCl solution per mL of leachate or 0.040 mL of NaOH solution per mL of leachate were used. Ammonium iron(II) sulphate hexahydrate ((NH_4_)_2_Fe(SO_4_)_2_, 99% w/w) and iron(III) chloride anhydrous (FeCl_3_, ≥97% w/w) received from Fisher Chemical were used as sources of ferrous and ferric ions, respectively. Hydrogen peroxide solution (37.6% w/w, Skat-Reactiv) was added as oxidant. Ultrapure water was used in all experiments.

### Landfill leachate characterization and physicochemical pretreatment

**Fig 1:**
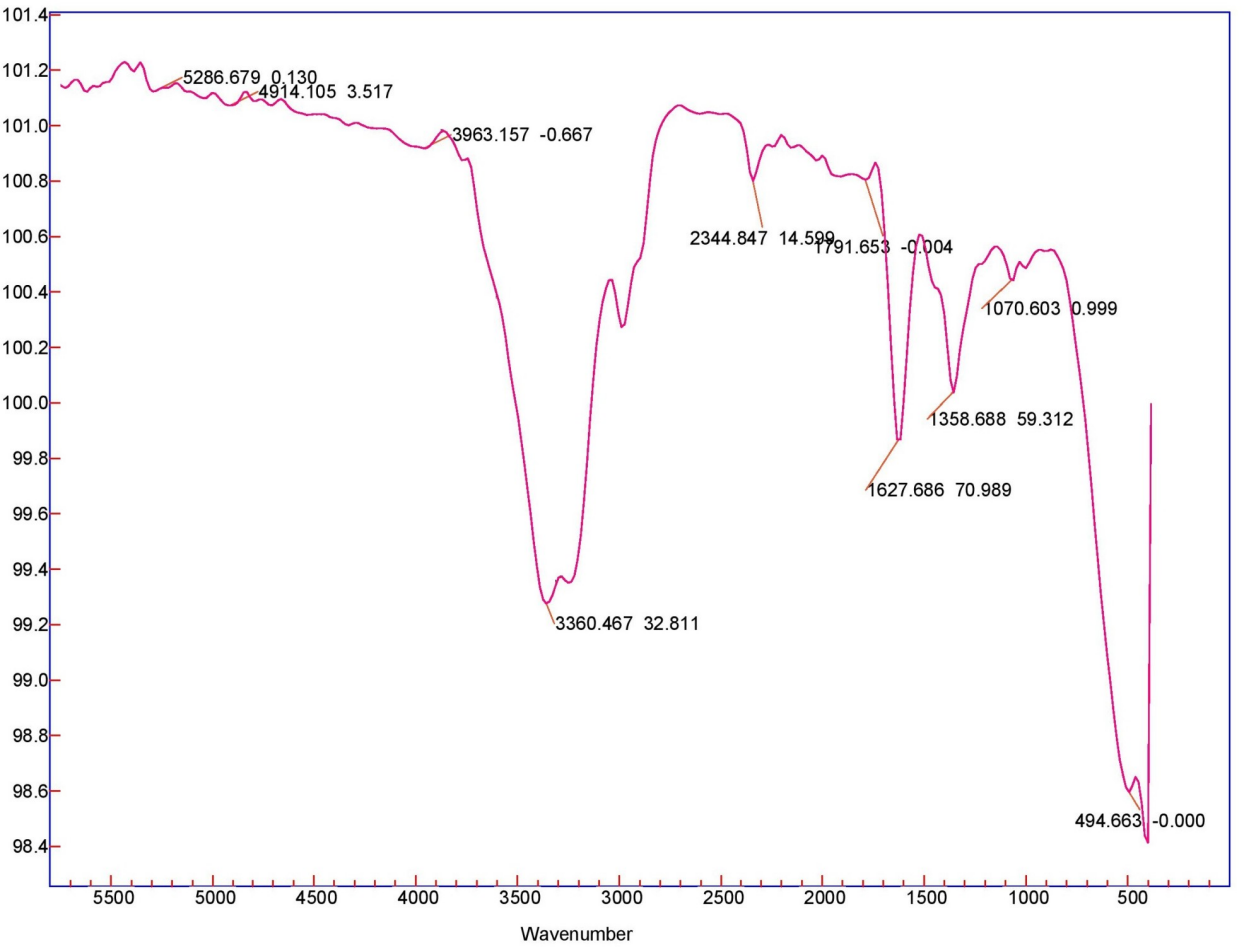
FT-IR analysis of the raw leachate.

The landfill leachate was sampled in batches over February-March 2018 from the MSW landfill of Nur-Sultan city (Kazakhstan). The access to the landfill site for the collection of leachate samples was permitted by the municipal solid waste management department of the Nur-Sultan city municipality. All necessary safety measures were taken prior to arrival at the MSW landfill site and landfill operators provided technical support for the collection of leachate samples from reservoirs. This leachate is classified as stabilized since the landfill age is more than 10 years [2, 4]. As shown in Table 1, its total carbon content was 5868±5% mg L^-1^, while 40–45% of that carbon was inorganic (2450±8% mg L^-1^). The total nitrogen concentration was 2905±3% mg L^-1^, 90% being inorganic in the form of ammonium. The iron content was 0.4±0.04 mg L^-1^, which was too low to substantially contribute to the photo-Fenton reactions. The pH of the raw leachate was 8.2. FT-IR analysis was conducted (Agilent Cary 600 Series) as shown in Fig 1 to qualitatively investigate the functional groups and its possible composition. Its interpretation is shown in Table 2 [33–36].

**Table 1:** Landfill leachate (raw) characterization.

Parameters	Range
TC (mg L^-1^)	5868 ± 293
TIC (mg L^-1^)	2450 ± 196
TOC (mg L^-1^)	3227 ± 252
TN (mg L^-1^)	2905 ± 87
TSS (g L^-1^)	0.17 ± 0.02
TS (g L^-1^)	13.5 ± 1.7
pH	8.2 ± 0.1
Conductivity (mS cm^-1^)	32.1 ± 0.4
N-NH_4_^+^ (mg L^-1^)	2615 ± 99
Chloride (mg L^-1^)	3316 ± 60
Sodium (mg L^-1^)	3379 ± 124
Potassium (mg L^-1^)	2049 ± 88
Magnesium (mg L^-1^)	236 ± 47
Calcium (mg L^-1^)	156.5 ± 29.4
Iron (mg L^-1^)	0.4 ± 0.04
Nitrate (mg L^-1^)	58.9 ± 1.2
Zinc (μg L^-1^)	512 ± 29.2
Chromium (μg L^-1^)	670 ± 16.8
Lead (μg L^-1^)	4.6 ± 1.1
Cadmium (μg L^-1^)	15.5 ± 2.5
Cobalt (μg L^-1^)	113 ± 17
Copper (μg L^-1^)	47 ± 12.5
Mercury (μg L^-1^)	1.2 ± 1

**Table 2:** The description of FT-IR analysis of the raw leachate.

Wavelength (cm^-1^)	Vibration	Functional group or component
3963	C-H stretch	Aliphatic methylene
3360	O–H stretch	Bonded and non-bonded hydroxyl groups and water
	≡C-H stretch	Alkyne group
2900	C-H stretch	Asymmetric and symmetric methyl and methylene groups
2344–2020	C-N	Cyanides (nitriles), cyanates, isocyanates, thiocyanates, and diazo compounds
1791	C = O	Acid (acyl) halide, aryl carbonate, open-chain acid anhydride, five-membered ring anhydride
1628	C = C stretch	Alkene and aromatic ring
	C = O stretch	Carboxylate and amide I
	O-H bend	Absorbed water
	N-H in bend	Amines
1358	C-N stretch	Aromatic secondary and tertiary amine
	N-O stretch	Nitrate
1070	C-O stretch	Alcohol and ether
	S–O stretch	Inorganic sulphates
1055–1020	Si-O-Si, Si-O stretch	Silica and clay minerals
	C-F	Fluoride
508	P-O	Inorganic phosphate
	C-Br	Bromide

Due to the high total solids (TSs) content, the raw leachate was left for a sufficient time for solids to precipitate before the experiments. Suspended particles can hinder the access of light to the reaction medium, as they can diffuse light and reduce UV transmittance of water [37]. Jamil et al. [38] studied COD and TSS (Total Suspended Solids) removals from a paper mill effluent, which was highly contaminated with TSS (5950 mg L^-1^), by using different AOPs. The photo-Fenton process resulted in high COD (79.6%) and TSS (96.6%) removals at a pH 3 with 0.5 g L^-1^ Fe(II) and 1.5 g L^-1^ H_2_O_2_ after 45 min of treatment.

Bouasla et al. [39] observed a decrease in the decolorization efficiency and the rate constant of azo dye Acid Yellow 99 (AY99) degradation in the presence of inorganic anions (Cl^-^ and SO_4_^2-^) and cations (Na^+^, Ca^2+^, Cu^2+^, Mn^2+^, Zn^2+^ and Mg^2+^) in the aqueous solution. This inhibition may be due to a complexation and a radical scavenging. Deng et al. [40] reported that nitrate did not significantly affect Fenton oxidation, sulfate slightly inhibited COD removal, whereas chloride considerably inhibited COD removal in Fenton treatment of a landfill leachate. The inhibiting effect may be due to scavenging of •OH and competition of chloride with H_2_O_2_ to form Fe(III) complexes, resulting in re-distribution of iron species and reduction in their reactivity. Machulek et al. [41] found that a pH control at 3 throughout the process prevents the inhibition of the photo-Fenton process by chloride ion even for a complex mixture of components. The photolysis of Fe(OH)^2+^ to Fe^2+^ and the formation of •OH are relatively efficient, even in the presence of high concentrations of chloride ions in the pH range of 3–3.3. At pH 2, photolysis of Fe^3+^ in the presence of chloride ions leads to almost exclusive formation of the less-reactive Cl_2_^-^• radical anion. Scavenging of the hydroxyl radical by chloride ion becomes important at pH 2 or below. Liu et al. [42] discovered that potassium ions accelerate the photo-Fenton process, while sodium, rubidium, and cesium cations decline the process.

Preliminary experiments showed that the inorganic carbon contained inhibited the photo-Fenton process by scavenging hydroxyl radicals [43]. Moreover, ammonia present at concentrations up to about 2000 mg L^-1^ in leachate can be toxic to microorganisms and inhibit biological treatment processes [4]. As a result, a pretreatment process was applied in two stages [44]: firstly, ammonia was eliminated by applying air stripping for 24 hours at pH = 12, and subsequently inorganic carbon was removed by regulated the leachate pH to 5. By using air stripping, undesired substances in the liquid can be removed with the carrier gas, commonly air [45]. Ammonia (a highly water-soluble gas) removal by air stripping is highly dependent on pH, and it is necessary to adjust the pH to 10 or more before the stripping. The ammonia stripping is based on the following reaction [46]:

$${NH}_{4}^{+}⇌{NH}_{3}+H^{+}$$ (11)

Leachate usually contains both inorganic-nitrogen compounds (ammonia, ammonium, nitrite, and nitrate) and organic-nitrogen compounds. Nitrification (the oxidation of ammonia to nitrate) and denitrification (the reduction of nitrate to nitrogen gas) processes are two main reaction pathways in the natural nitrogen cycle [47].

After acidification (the second stage of the pretreatment), the equilibrium, in which the solids remained suspended due to the dispersant action of the additives, was disrupted, which led to the rapid precipitation of suspended solids. Partial color removal was also observed, most likely due to the adsorption of pigments on the surface of the solids [48]. The pretreatment of the raw leachate led to 100% removal of inorganic carbon, 98.7% ammonia removal and 20% removal of organic contaminants, as it is shown in Table 3.

**Table 3:** Pretreated leachate characterization.

Parameters	Range
TOC (mg L^-1^)	2600 ± 200
TN (mg L^-1^)	290 ± 40
pH	4.9 ± 0.1
Conductivity (mS cm^-1^)	59.2 ± 0.2
N-NH_4_^+^ (mg L^-1^)	35 ± 10
Potassium (mg L^-1^)	2042 ± 102
Magnesium (mg L^-1^)	151 ± 25
Calcium (mg L^-1^)	80 ± 8
Iron (mg L^-1^)	0.33 ± 0.04
Zinc (μg L^-1^)	358 ± 30
Chromium (μg L^-1^)	586 ± 14
Lead (μg L^-1^)	2.3 ± 1
Copper (μg L^-1^)	29 ± 3

Prior to the photochemical treatment, the pretreated leachate was mixed with tap water at a ratio of 5/12 (volume basis) [44]. The initial carbon concentration of the pretreated diluted leachate was in the range of 1200±100 mg L^-1^ with all carbon coming from organic compounds, while its total nitrogen was 150±24 mg L^-1^ and pH in the range of 5.1–5.3.

### Experimental procedure

**Fig 2:**
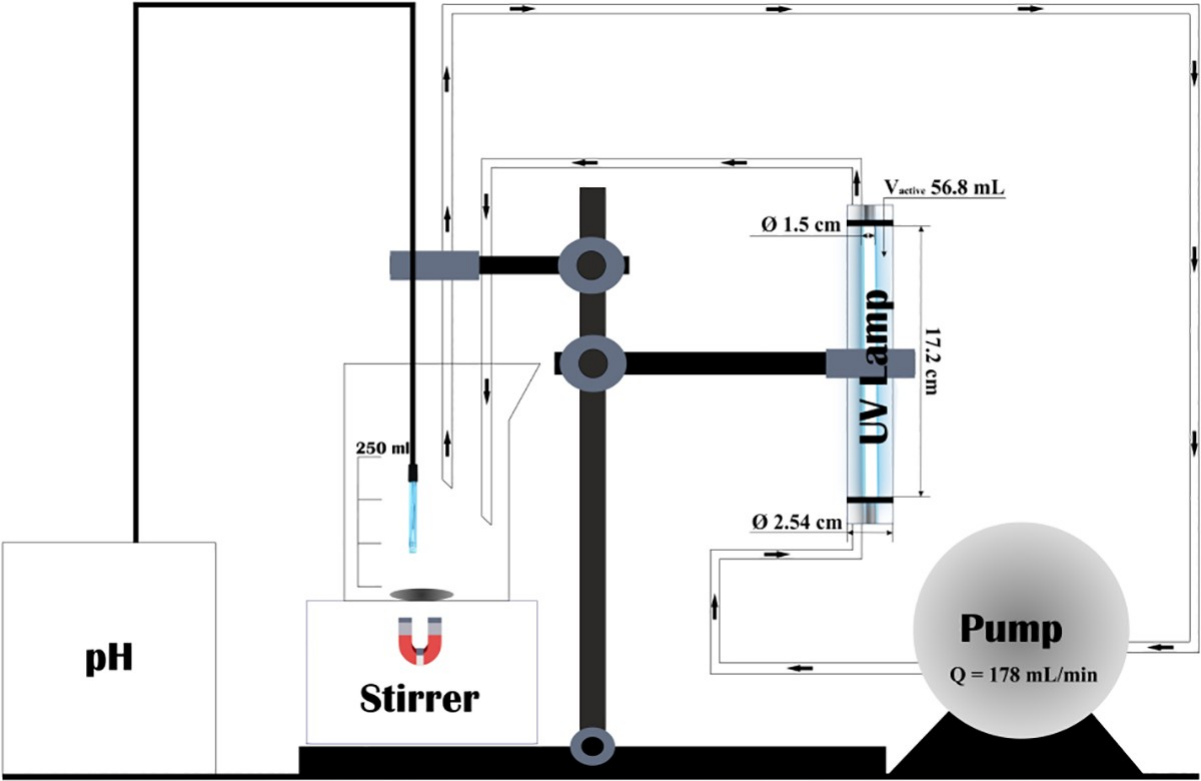
The experimental setup.

All experiments were carried out using the apparatus shown in Fig 2.

The total volume of the treated solution was 250 mL. An annular photoreactor with 56.8 mL of active volume operated in batch recycle mode was used for photochemical experiments. The solution was continuously pumped at the rate of 178 mL min^-1^ through the photoreactor using a peristaltic pump (Heidolph 5006) [44]. An Osram lamp with 6 W power, located inside the photoreactor, emitted UV irradiation of 254 nm.

The part of the solution that was not exposed to irradiation was constantly stirred with a magnetic stirrer (Bibby Scientific, United Kingdom). The start of the experiment was considered with the switch-on of the UV lamp. The pH was measured throughout the experiments using a Mettler Toledo LE409 Electrode immersed in the solution. The duration of experiments was 2 hours. Aliquots from the reaction mixture were periodically taken and sent directly to the analysis. A Vitlab 1000 μL automatic pipette was used for sampling.

The leachate after the full pretreatment contained all carbon in the organic form with an initial concentration in the range of 1200±100 mg L^-1^, while the total nitrogen was 150±24 mg L^-1^ and the pH in the range of 5.1–5.3. The experiments were conducted using different initial dosages of Fe(II) (200–600 ppm), Fe(III) (300–700 ppm) and H_2_O_2_ (0–9990 mg L^-1^).

### Analytical methods

The treatment efficiency was assessed through pH measurements, TC/TIC/TN (Total Carbon/Total Inorganic Carbon/Total Nitrogen) analyses, and color change. The TC/TIC/TN analyses were performed using the Multi N/C 3100 instrument by Analytik Jena AG (Germany), whereas color change was attended using a photoLab® 6000 series UV-Vis spectrophotometer. Ions in the leachate were measured by ion chromatography (IC, 930 Compact IC Flex, Metrohm). Metals in the leachate were measured using ICP-MS iCAP RQ supplied by Thermo Scientific. Conductivity was measured with a digital EC meter (Five Easy™ FE30). Leachate TSS (Total Suspended Solids) were measured by filtering under vacuum 20 mL of sample through a 1.2 μm glass fiber filter paper, and TS (Total Solids) measurement was conducted by placing 45 mL of sample in a beaker heated at 105°C in an oven for 24 h [44]. All measurements were conducted at least twice for three samples. The FT-IR spectra of the raw leachate was recorded using Agilent Cary 600 Series. All samples were filtered before analysis using Agilent Captiva premium syringe filters with a 0.45 μm regenerated cellulose (RC) membrane.

The removal efficiency was calculated according to Eq (12):

$$Removal\;efficiency\;\left(\%\right)=\left(1-\frac{C_{t}}{C_{0}}\right)\times 100,$$ (12)

where *C*_*t*_ is the concentration after time *t* and *C*_0_ is the initial concentration. Preliminary experiments run in triplicates showed that the average error was up to 4% (as one standard deviation of the mean).

## Results and discussion

### Effect of inorganic carbon

Initially, the effect of the inorganic carbon presence on the photochemical oxidation of the partially pretreated leachate solution was examined, namely after the first step of pretreatment (ammonia removal). The experiments were conducted using 6660 mg L^-1^ of H_2_O_2_ and 400 ppm of Fe(II). The total initial carbon of the partially pretreated leachate was 3176 mg L^-1^ with 1541 mg L^-1^ coming from inorganic carbon (48.5% of total carbon). The initial pH of the partially pretreated leachate was equal to 10.1, and remained around 10 throughout the experiment. The leachate after full pretreatment contained only organic carbon (1342 mg L^-1^) and it had pH 5.3, which was reduced to 4.5 during the experiment. Iron precipitates as hydroxide when the value of pH is higher than 6, which decreases the available iron in the solution and the process efficiency [49]. Moreover, hydrogen peroxide is decomposed under alkaline conditions and the production of hydroxyl radicals is decreased [50]. Since acidic conditions have been reported to be crucial to promote the photo-Fenton oxidation in the great majority of relevant studies, the pH of the solution was not re-adjusted after the removal of the inorganic carbon [51].

**Fig 3:**
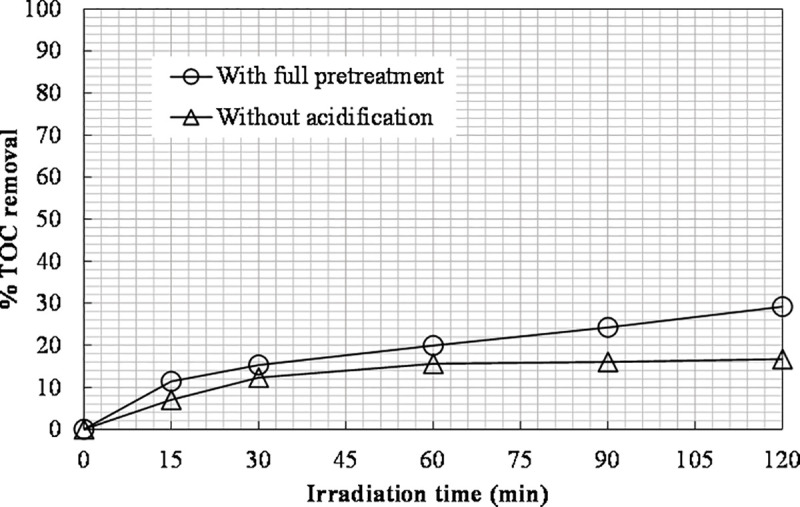
The effect of inorganic carbon on TOC removal ([Fe(II)]_0_ = 400 ppm, [H_2_O_2_]_0_ = 6660 mg L^-1^).

The results obtained are shown in Fig 3. It was clear that the presence of inorganic carbon inhibited the organic carbon removal in the photo-Fenton process since the decrease in the total organic carbon of the partially pretreated leachate (without acidification) finally achieved was 17%. 29% TOC removal was achieved along with improved color change and complete removal of TIC in the case of the completely pretreated leachate.

Inorganic carbon, such as carbonate CO_3_^2-^ and bicarbonate HCO_3_^-^, can affect the overall photochemical reaction rate through reacting with the generated •OH [13, 43]. Reducing the pH to acidic conditions eliminates this issue, since CO_3_^2-^ and HCO_3_^-^ in combination with H^+^ form unstable carbonic acid (H_2_CO_3_), which is then decomposed to CO_2_ and H_2_O [13].

The TIC removal of the partially pretreated leachate was zero, as the photo-Fenton process has no impact on the inorganic carbon content. The TN value (remaining nitrogen after pretreatment) was unchanged during both experiments, 180±2% mg L^-1^. The results obtained confirmed the necessity for a second stage of pretreatment to remove the inorganic carbon present in the leachate, namely the pH adjustment to 5.

### Effect of Fe(II) concentration

**Fig 4:**
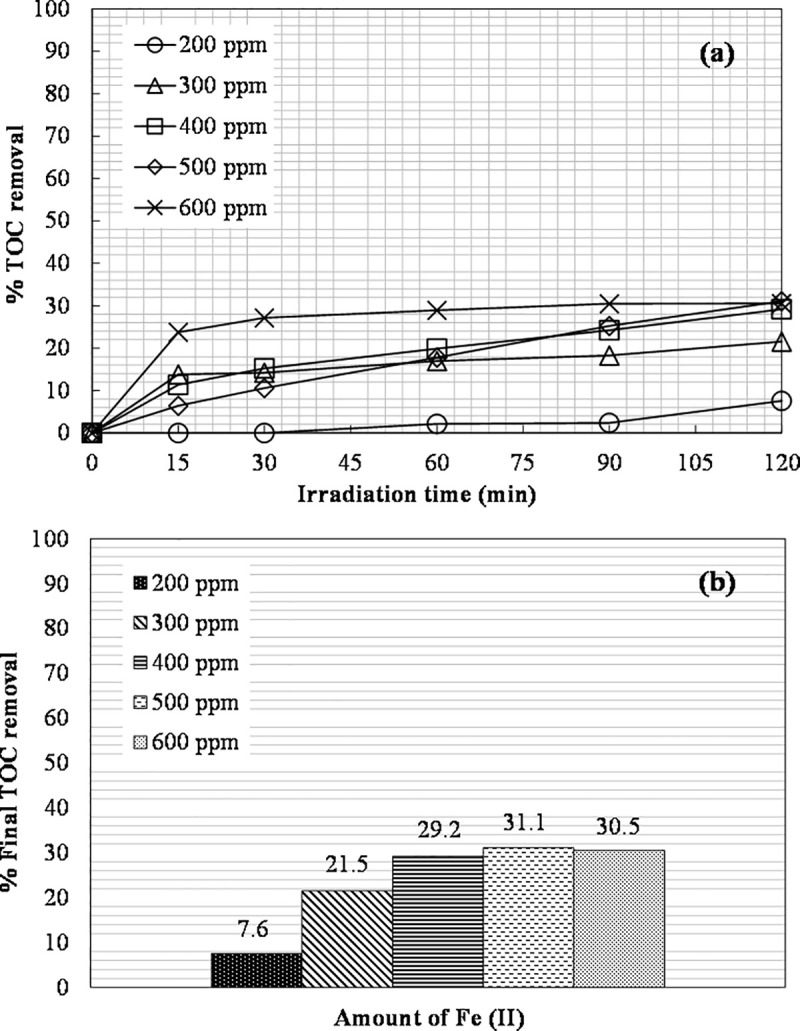
The effect of Fe(II) concentration on: (a) TOC removal vs. irradiation time; (b) The final TOC removal achieved ([H_2_O_2_]_0_ = 6660 mg L^-1^).

The initial concentration of ferrous ions was varied to find the optimum one. The hydrogen peroxide concentration was kept constant for these tests at 6660 mg L^-1^. It was observed that the increase in Fe(II) concentration from 200 to 500 ppm increased the TOC removal from 8 to 31% (Fig 4). After further increasing to 600 ppm, the achieved percentage of TOC removal was 30.5%. The difference in TOC removal between 400 and 500 ppm of Fe(II) was only 2%, and considering that it is more cost-effective to use a smaller amount of reagent to keep low the formation of iron sludge [52], it was decided to use 400 ppm of Fe(II) in the next photo-Fenton experiments.

According to Deng and Englehardt [4], the removal of organic pollutants by photo-Fenton increases with increasing iron concentration, but further removal may become insignificant when the iron concentration is high. In addition, there is a limit to the concentration of iron that can be used due to the scavenging effect of excess iron on •OH (Eq 5).

**Fig 5:**
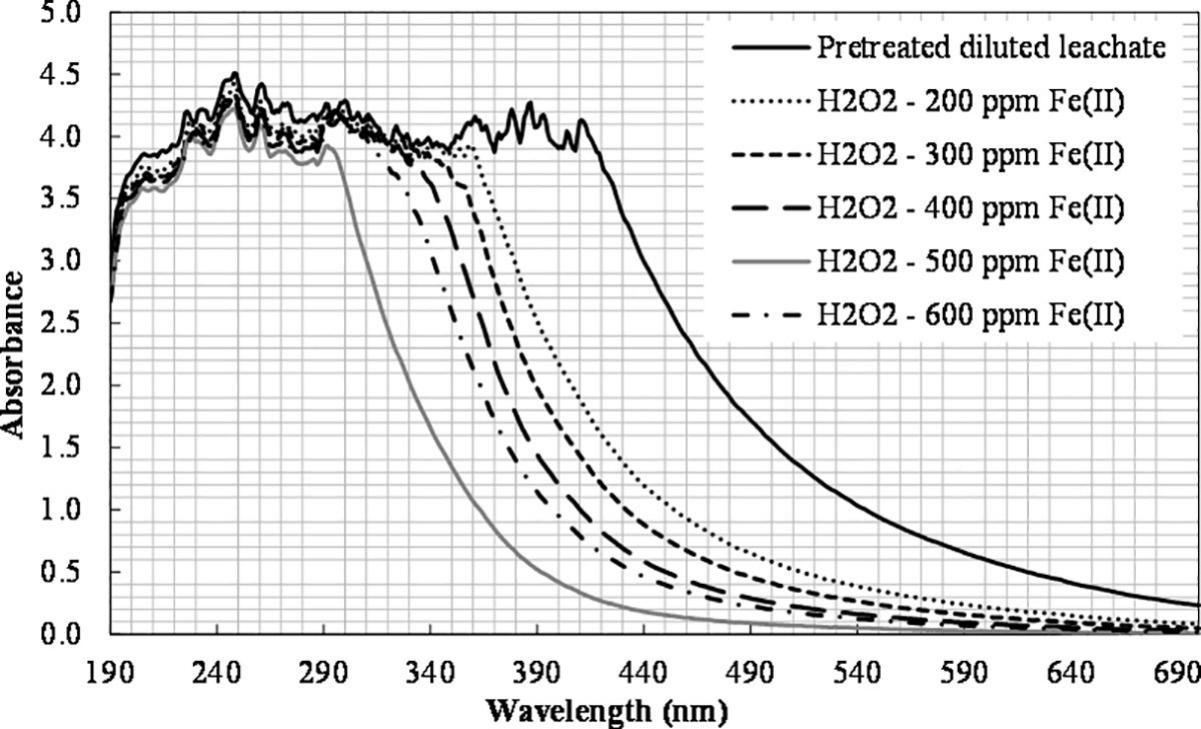
The effect of Fe(II) concentration on color change ([H_2_O_2_]_0_ = 6660 mg L^-1^).

The initial pH for tests with varying Fe(II) concentrations ranged from 5.2 to 5.3 and dropped to about 4.6 during the experiments, possibly due to the formation of organic acids during the process [53]. A color improvement after the photochemical treatment was also observed. The UV-Vis spectra indicating the color change are shown in Fig 5.

In old landfills, non-biodegradable substances predominate the organic fraction of the leachate [9] giving its dark color [54], while their molecular weight increases as leachate matures [55]. Large organic compounds are converted into smaller molecules during the treatment process, which leads to color improvement. Kim and Huh [32] also observed a 92% improvement in the color of a mature leachate through the Fenton process.

A dark Fenton experiment (without UV light) was conducted to compare the efficiency of the use of UV light. The experiment was carried out using 6660 mg L^-1^ of H_2_O_2_ and 400 ppm of Fe(II), and the achieved TOC removal was 21%, which was lower than the 29% obtained by the photo-Fenton process with the same amount of reagents. Applying ultraviolet light to the oxidation process improves the removal of contaminants [24].

### Effect of Fe(III) concentration

The effect of the concentration of ferric ions (instead of ferrous ions) on the removal of organic contaminants during the photo-Fenton-like process was studied using the same initial concentration of hydrogen peroxide (6660 mg L^-1^) and different initial dosages of Fe(III) in the range of 300 to 700 ppm.

**Fig 6:**
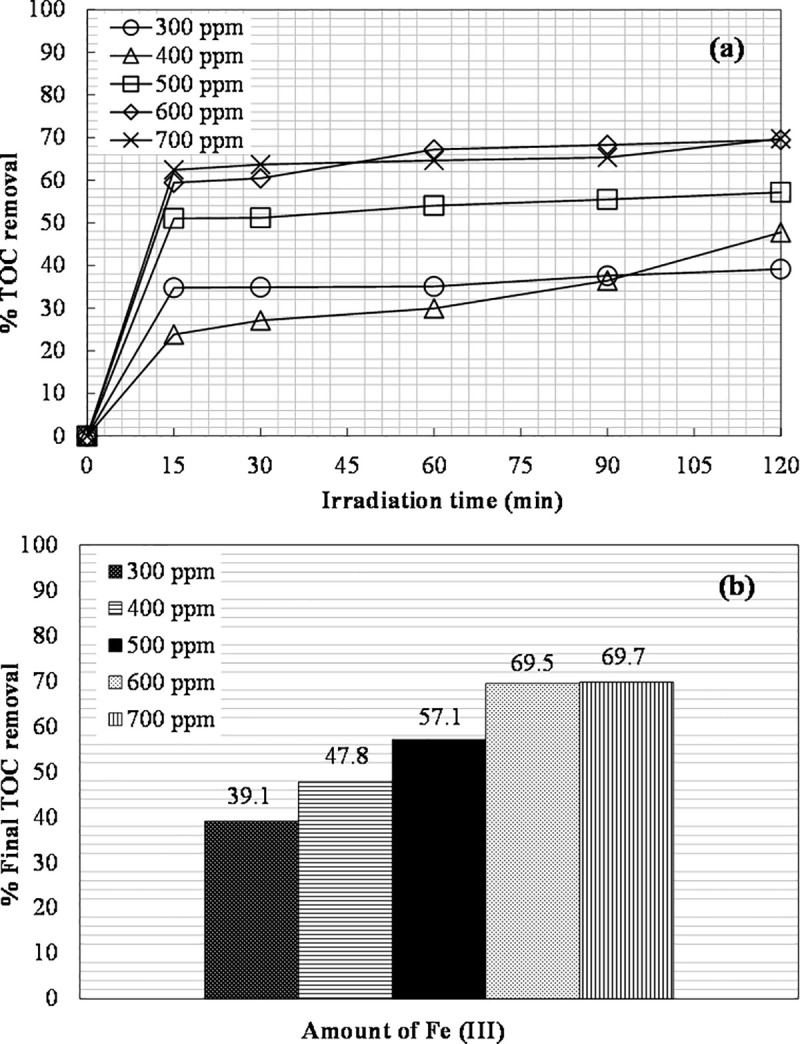
The effect of Fe(III) concentration on: (a) TOC removal vs. irradiation time; (b) The final TOC removal achieved ([H_2_O_2_]_0_ = 6660 mg L^-1^).

Replacing ferrous ions with ferric ions proved to be favorable for TOC removal, especially during the first 15 min. The results are shown in Fig 6.

The removal of TOC by the photo-Fenton-like method gradually increased from 39% to 69.5% with increasing the concentration of Fe(III) from 300 to 600 ppm. However, a further increase to 700 ppm did not result in a further improvement. Consequently, 600 ppm of Fe(III) was used for further photo-Fenton-like experiments. Fe(III) showed the same tendency as Fe(II); the process was favored by the increase in ferric ions concentration only up to a point, which was in agreement with the work of Deng and Englehardt [4].

Dark Fenton-like process (600 ppm of Fe(III) and 6660 mg L^-1^ of H_2_O_2_) was also studied for comparison and showed a lower TOC removal (63%) than the photo-Fenton-like process (69.5%).

**Fig 7:**
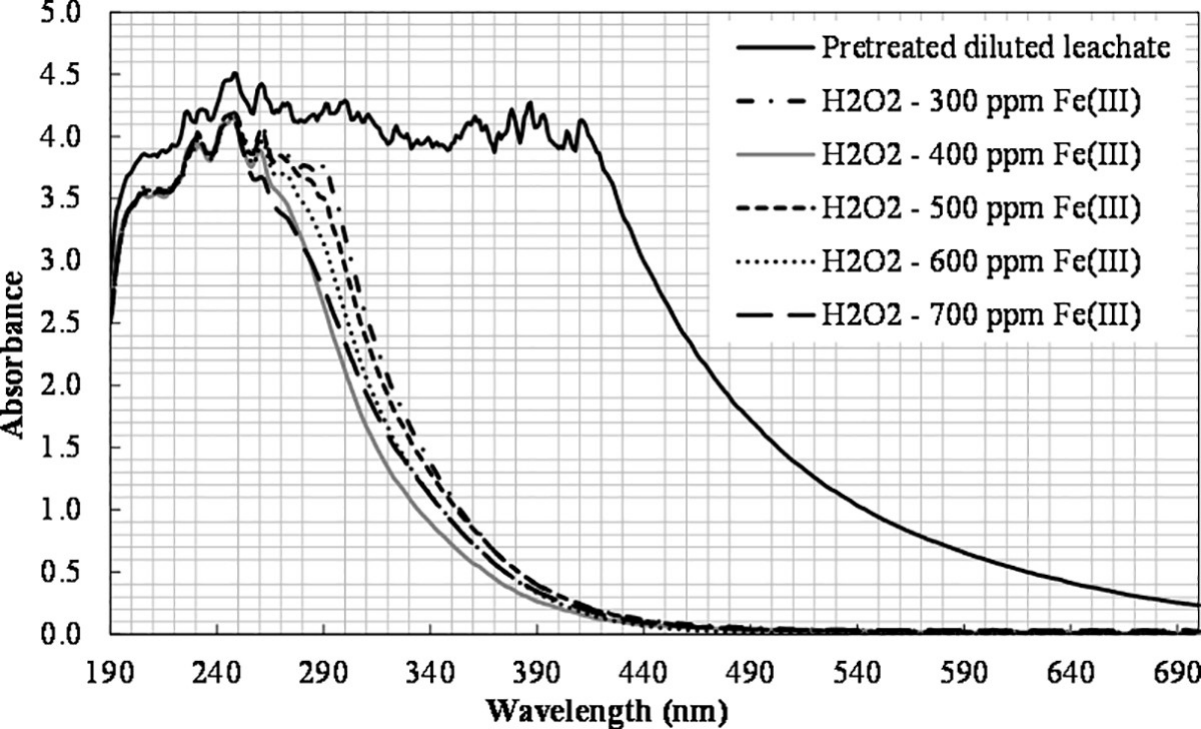
The effect of Fe(III) concentration on color change ([H_2_O_2_]_0_ = 6660 mg L^-1^).

Comparing the results with the photo-Fenton process, it is clear that the efficiency of TOC removal and color change (Fig 7) obtained in the photo-Fenton-like method was higher. Several studies have been conducted to compare both processes, and the results obtained are controversial. Deng and Englehardt [4] stated that the photo-Fenton-like process had a low rate of formation of hydroxyl radicals, while at the beginning of the photo-Fenton oxidation a very rapid formation of hydroxyl radicals was observed due to the higher rate constant in the photo-Fenton. Kim et al. [56] also reported that the photo-Fenton reaction had higher removal than the photo-Fenton-like reaction. Rivas et al. [57] found that similar removal efficiencies of organic matter were obtained with photo-Fenton and photo-Fenton-like processes. On the other hand, Rodríguez Narváez et al. [58] compared Fenton and Fenton-like processes for l-proline decomposition, and reported that the Fenton-like system showed the highest degradation. Bautista et al. [59] also found that the ferric ion (Fenton-like process) showed a comparable or better catalytic effect than the traditionally used ferrous ion (Fenton process) in experiments with both batch and continuous flow with real cosmetic wastewaters. According to Devi et al. [60], the higher efficiency of ferric ions can be attributed to the generation of hydroperoxyl radicals that convert ferric ions into ferrous ions much faster. The so generated ferrous ions react with hydrogen peroxide to form hydroxyl radicals. Ferric ions can also lead to ferrous ions and hydroxyl radicals by reacting with water molecules. The authors stated that although this sequence of reactions can also be observed in the case of the photo-Fenton treatment, the ratio of Fe^2+^/Fe^3+^ concentrations is crucial for the overall performance. The photo-Fenton-like process has higher concentration of ferric ions, which accounts for its higher efficiency. Gogate and Pandit [52] argued that the role, the underlying mechanism, and the equilibrium concentration of ferrous and ferric ions are complex and unclear in detail.

### Effect of H_2_O_2_ dosage

**Fig 8:**
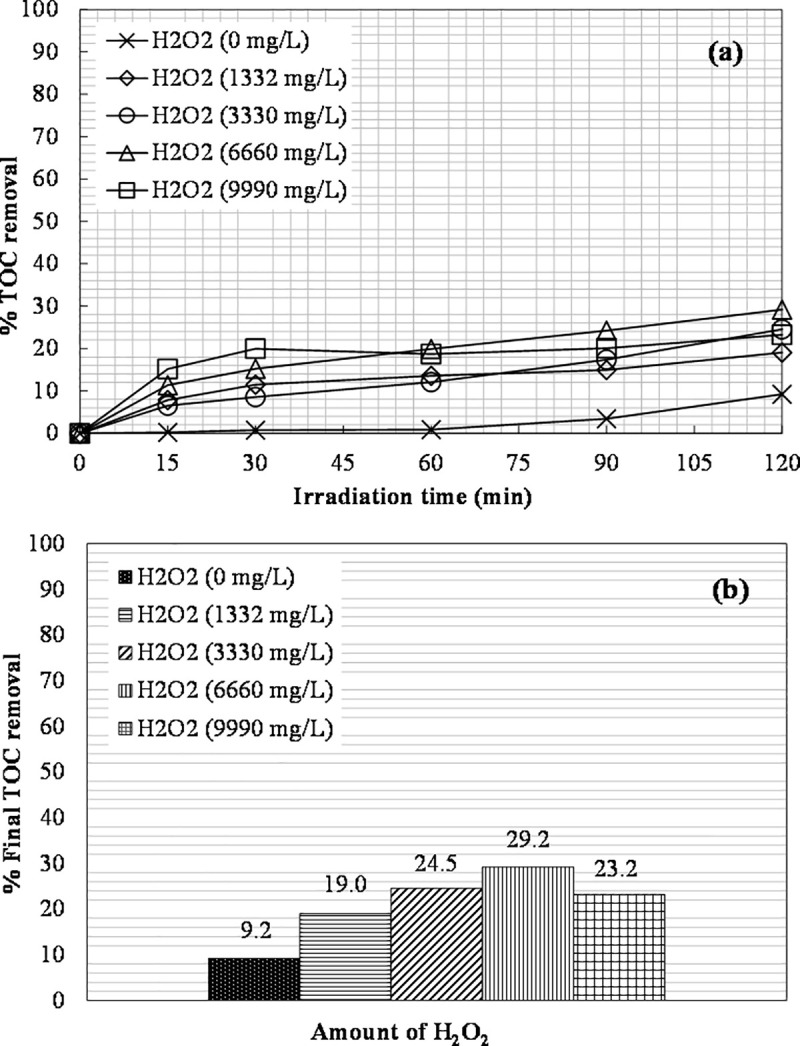
The effect of H_2_O_2_ dosage on: (a) TOC removal vs. irradiation time; (b) The final TOC removal achieved ([Fe(II)]_0_ = 400 ppm).

**Fig 9:**
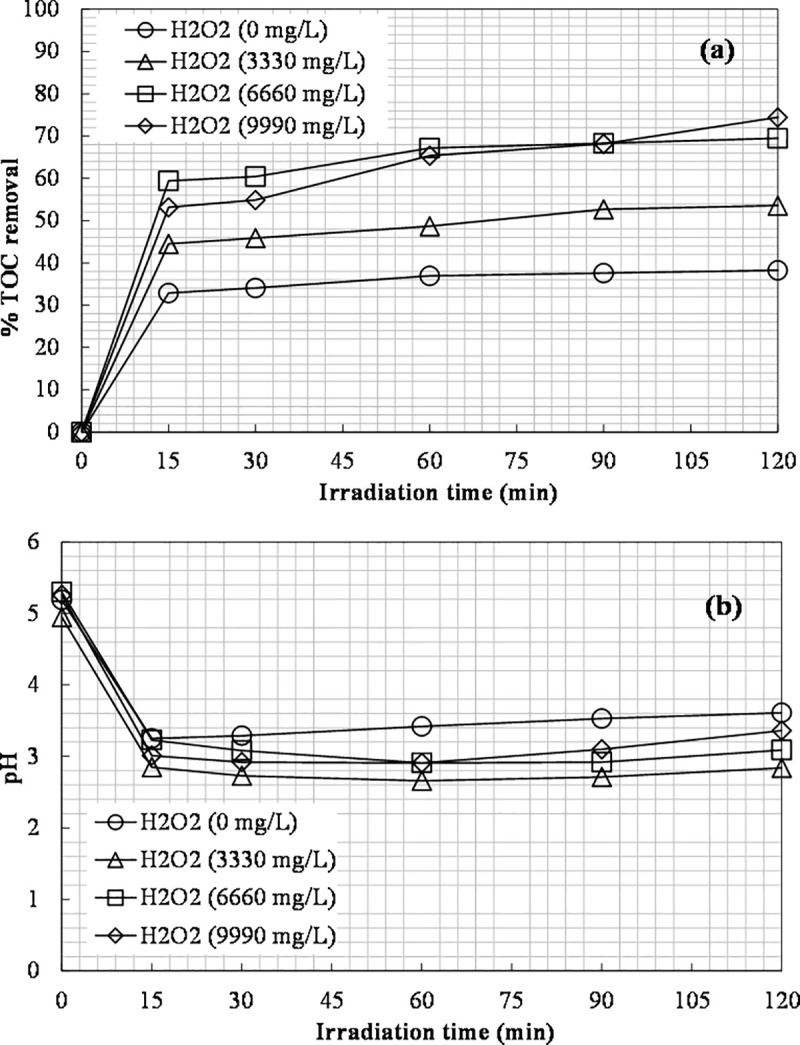
The effect of H_2_O_2_ dosage on: (a) TOC removal; (b) pH ([Fe(III)]_0_ = 600 ppm).

The effect of hydrogen peroxide dosage was studied by keeping constant the initial iron concentration. Experiments were performed for 0, 1332, 3330, 6660, and 9990 mg L^-1^ of H_2_O_2_. 400 ppm of Fe(II) were used for the photo-Fenton process, while 600 ppm of Fe(III) for the photo-Fenton-like process. The results obtained are shown in Figs 8 and 9. The most favorable concentration of H_2_O_2_ for carbon removal was 6660 mg L^-1^ leading to 29% TOC removal with 400 ppm of Fe(II) and 69.5% TOC removal with 600 ppm of Fe(III).

A higher initial concentration of hydrogen peroxide leads to a higher removal of TOC [4]. A further increase in the dosage of hydrogen peroxide to 9990 mg L^-1^ in the photo-Fenton-like process led to a slight increase in TOC removal (74.4%) compared to 6660 mg L^-1^ of H_2_O_2_ (69.5%). Thus, it was more practical to use 6660 mg L^-1^ of H_2_O_2_ for the next experiments. In the photo-Fenton process, an increase in the H_2_O_2_ dosage from 6660 to 9990 mg L^-1^ resulted in a decrease in the removal of TOC from 29% to 23%. Singh et al. [61] also observed a decrease in the efficiency of methylene blue dye degradation with an excess of H_2_O_2_ using ZnO–Fe_3_O_4_ nanocomposites under UV radiation. The methylene blue degradation efficiency decreased from 92.3 to 59.7% with an H_2_O_2_ increase from 0.01 to 0.5%. Hydrogen peroxide in excess can act as hydroxyl radical scavenger itself [4, 61]. An overdose of H_2_O_2_ can lead to reaction with •OH radical and the formation of the perhydroxyl radical HO_2_•, which is an undesired product [62]. The scavenging effect is shown in Eq (4).

The pH during photo-Fenton experiments dropped from about 5.3 to 4.6 in all cases, except for the experiment without hydrogen peroxide, where the pH slightly fluctuated from the initial value. During the photo-Fenton-like experiments (Fig 9B), the initial pH value was around 5.2, and it dropped gradually as the process progressed, possibly due to the conversion of organic carbon into organic acids; then, the pH value rose again as the organic acids began to decompose slowly to CO_2_ leaving the solution [53]. The TN value was unchanged throughout all experiments and remained in the range of 130–180 mg L^-1^, depending on the value achieved after the pretreatment step. The •OH radicals generated during photo-Fenton cannot oxidize ammonia despite their rather strong oxidizing ability [4].

### Effect of pH adjustment

**Fig 10:**
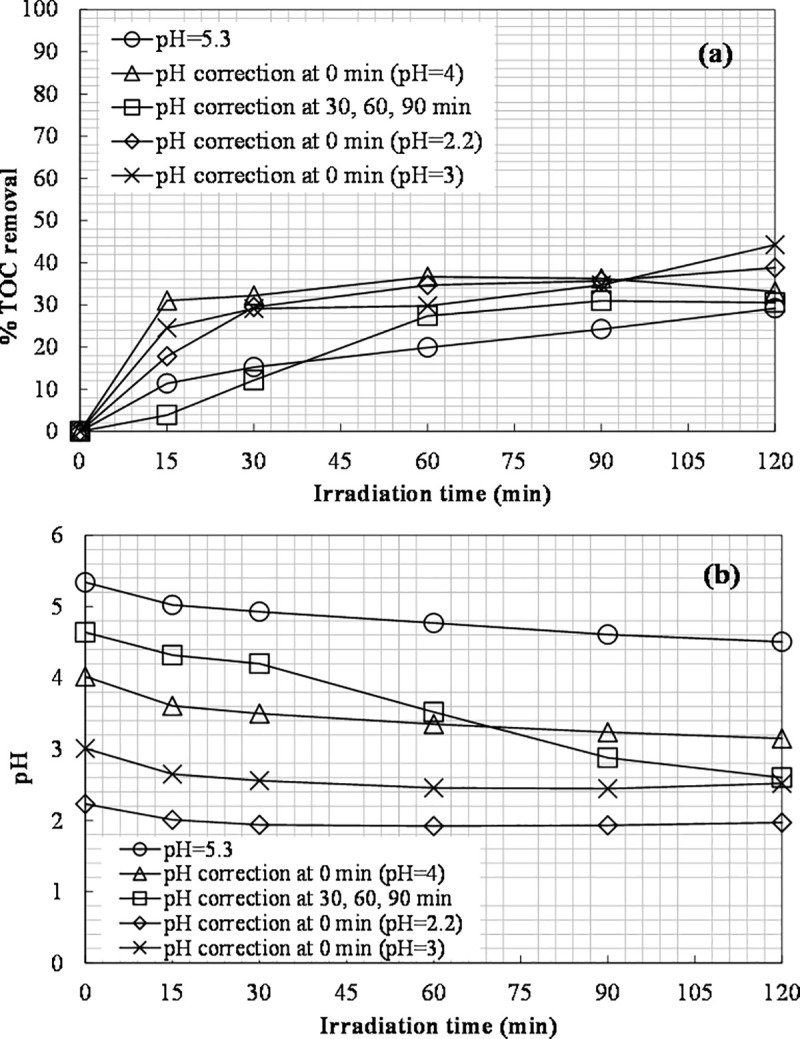
The effect of pH adjustment on: (a) TOC removal; (b) pH ([Fe(II)]_0_ = 400 ppm, [H_2_O_2_]_0_ = 6660 mg L^-1^).

Finally, the initial pH value was varied to examine the influence on the photo-Fenton process, since pH may have a considerable impact on the efficiency of the treatment [27]. Specifically, the pH effect on the decomposition of the leachate organic carbon was investigated using 6660 mg L^-1^ of H_2_O_2_ and 400 ppm of Fe(II). The TOC removal and pH values obtained are shown in Fig 10.

The initial pH of the pretreated leachate prior to any modification was 5.3. Firstly, the pH value was adjusted to 4 at the start of the experiment, which had as a result an increase of the final TOC removal from 29% (base experiment; no pH adjustment) to 33%. In the second run, the same amount of hydrochloric acid was added to the solution gradually, namely at 30, 60 and 90 minutes, and 31% of TOC removal was obtained.

**Fig 11:**
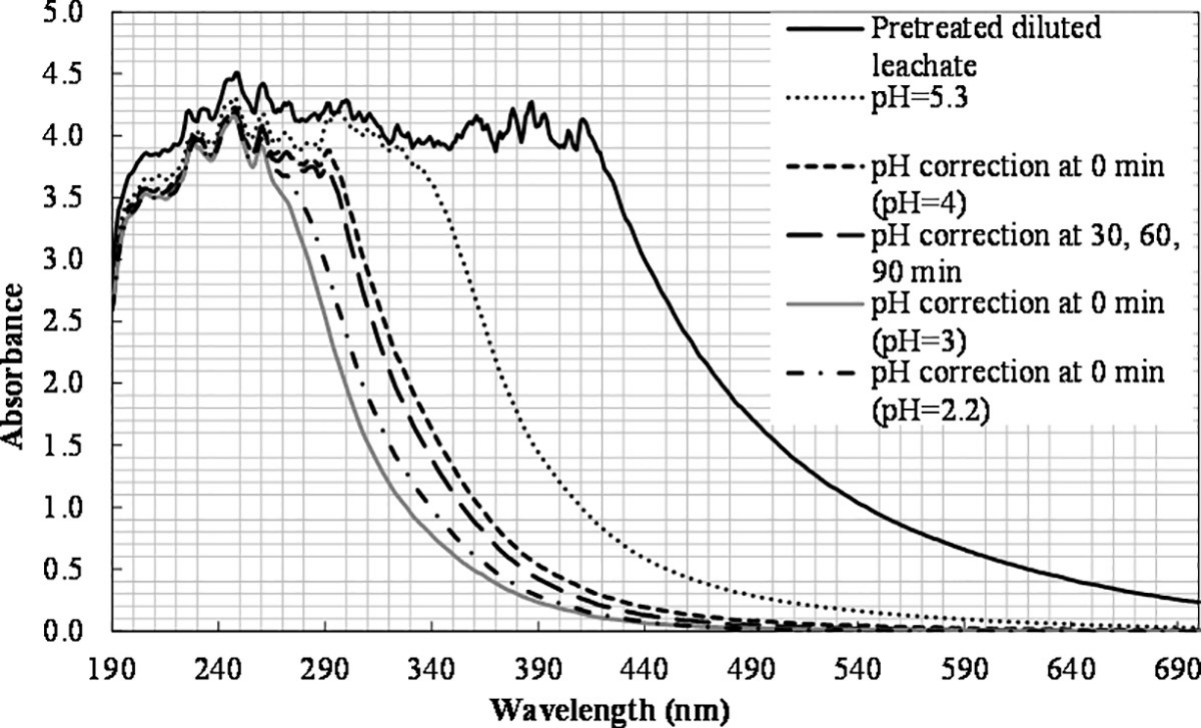
The effect of pH adjustment on color change ([Fe(II)]_0_ = 400 ppm, [H_2_O_2_]_0_ = 6660 mg L^-1^).

Furthermore, the initial pH value was varied from 2.2 to 5.3 to find the optimum value. The TOC conversions at pH 2.2 and 4 were equal to 39% and 33%, respectively. The highest TOC removal (44%) and color change corresponded to pH 3, while the lowest to pH 5.3 (29%). The nitrogen contained (165±22 mg L^-1^) was not affected in all cases. The color change during these experiments is shown in Fig 11.

The observation that the highest TOC removal took place at pH 3 is in agreement with the findings of other studies [24, 63–65]. The [Fe(OH)]^2+^ ion is of great importance for the photo-Fenton process, and it is formed at pH 2.8–3.5 [24]. The ferrous ions are unstable and can be transformed to ferric ions, forming complexes with hydroxyl at pH values above 4. Additionally, the decline in the process efficiency for pH values higher than 3 is associated with the dissociation and auto-decomposition of H_2_O_2_, which reduces its oxidizing ability [49, 50]. [Fe(H_2_O)]^2+^ forms at lower pH values (below 2) and reacts slowly with H_2_O_2_ [66]_._ Moreover, at a lower pH, the scavenging effect of H^+^ on hydroxyl radicals becomes more significant and slows down the process [67]. The reaction between Fe^3+^ and hydrogen peroxide can be inhibited at extremely low pH [68].

**Fig 12:**
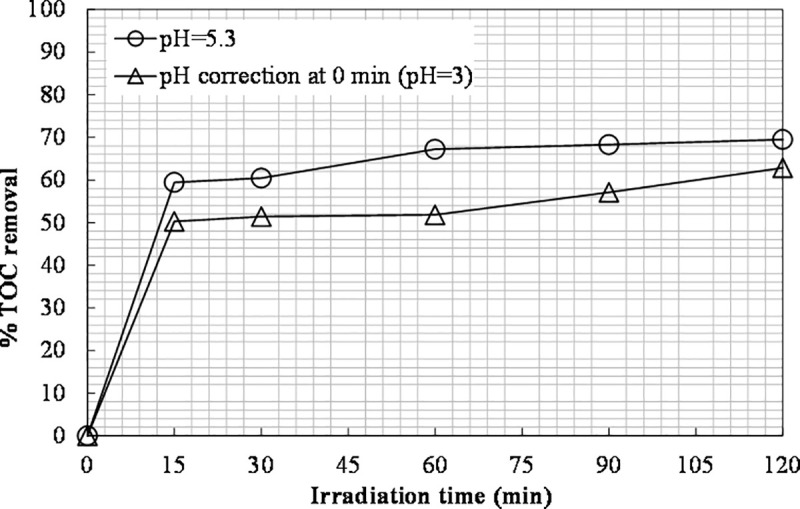
The effect of pH adjustment on TOC removal ([Fe(III)]_0_ = 600 ppm, [H_2_O_2_]_0_ = 6660 mg L^-1^).

Since better results were obtained by adjusting the initial pH of the solution in Fe(II) experiments, the most beneficial value of pH (pH adjustment to 3) was tested for Fe(III). 6660 mg L^-1^ of H_2_O_2_ and 600 ppm of Fe(III) were used. The TOC removal results are shown in Fig 12.

The final TOC obtained was 63%, lower than the one achieved without pH adjustment in the case of Fe(III) (69.5%). This result is consistent with the one reported by Kim et al. [56], namely the most favorable pH value for the photo-Fenton process is not necessarily the most beneficial one for the photo-Fenton-like process.

**Fig 13:**
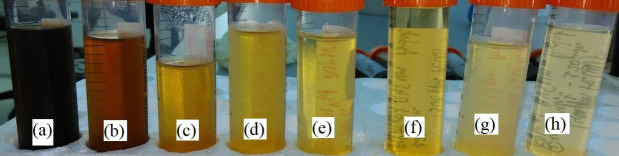
Color change ([H_2_O_2_]_0_ = 6660 mg L^-1^): (a) pretreated leachate; (b) UV-H_2_O_2_ process; (c-g) photo-Fenton process in the range of 200–600 ppm of Fe(II); (h) photo-Fenton-like process with 400 ppm of Fe(III).

The color change of the leachate itself after each process applied is shown in Fig 13 and provides a simple indication of the success of the treatment since the complex composition of leachates along with the variety of the oxidation intermediates, constitute unpractical the detailed detection and quantification of the species present in the wastewater throughout the treatment. Although not measured in this work, biodegradability enhancement [69, 70] and biotoxicity decrease [71, 72] of leachates have been reported during the photo-Fenton treatment.

## Conclusions

In this work, the efficiency of photo-Fenton and photo-Fenton-like processes was examined in the treatment of a leachate collected from the MSW landfill in Nur-Sultan, the capital city of Kazakhstan. The effectiveness of each process was evaluated by means of carbon removal and color change. The main conclusions are:

- A pretreatment stage including air stripping and pH adjustment was required for ammonia and inorganic carbon removal before the application of the photochemical process. The inorganic carbon removal was critical for the success of next steps because it was indirectly shown to act as hydroxyl radicals scavenger. Specifically, the TOC removal achieved was increased from 17% to 29% after removing completely the inorganic carbon from the leachate.
- The most favorable concentrations of H_2_O_2_ and Fe(II) for carbon removal were 6660 mg L^-1^ and 400 ppm, respectively, resulting in 29% TOC removal, while ammonia was not affected.
- The photochemical treatment was enhanced by replacing ferrous ions with ferric ions. Using 600 ppm Fe(III) instead of Fe(II) in the presence of 6660 mg L^-1^ of H_2_O_2_ and UV light had as a result an increase in the TOC removal from 27% to 69.5% and improved the leachate color. The most favorable concentration of H_2_O_2_ for carbon removal with Fe(III) was also 6660 mg L^-1^, as it was in the case with Fe(II).
- The most favorable initial pH value for photo-Fenton was found to be 3 as it resulted in 44% of TOC removal. In the case of Fe(III) changing the initial pH value did not improve the process.
- After the application of the two-steps pretreatment process followed by the most efficient photochemical process among the ones tested, the following removals were achieved: TC: 93.4%, TIC: 100%, TOC: 88.7%, TN: 96.5%, Color: 98.2% (at 450 nm).

## Supporting information

S1 File Total organic carbon removal and color change for the photochemical treatment of a landfill leachate using UV light, H2O2, and ferrous or ferric ions. (XLSX)
